# Assessment of Circulating Nucleic Acids in Cancer: From Current Status to Future Perspectives and Potential Clinical Applications

**DOI:** 10.3390/cancers13143460

**Published:** 2021-07-10

**Authors:** Gabriella Cirmena, Martina Dameri, Francesco Ravera, Piero Fregatti, Alberto Ballestrero, Gabriele Zoppoli

**Affiliations:** 1 Department of Internal Medicine, University of Genoa, 16132 Genoa, Italy; gabriella.cirmena@unige.it (G.C.); martina.dameri@hsanmartino.it (M.D.); francesco.ravera@edu.unige.it (F.R.); aballestrero@unige.it (A.B.); 2 U.O.C. Clinica di Chirurgia Senologica, Department of Surgery, IRCCS Ospedale Policlinico San Martino, 16132 Genoa, Italy; piero.fregatti@unige.it

**Keywords:** liquid biopsy, biomarkers, cell-free DNA, circulating tumor DNA, precision oncology

## Abstract

**Simple Summary:**

Liquid biopsy, defined as the family of methods aimed at identifying tumor biomarkers through noninvasive analysis of body fluids, is gaining more and more interest in the clinical setting as it represents a minimally invasive and cheap approach for the screening of cancer samples of different types. Cell-free nucleic acids represent one of the most promising biomarkers obtained from liquid biopsy, with actual and potential applications for various clinical purposes. However, standardized pre-analytical procedures as well as best-practice, highly reproducible extraction processes and quality control methods are still lacking, making it difficult to support the full implementation of cell-free nucleic acids assessment in routine clinical practice. Furthermore, the clinical utility of these biomarkers still appears to be relatively limited and focused on specific purposes. In this review, we analyze pre-analytical and analytical factors concerning cell-free nucleic acids, with a focus on cell-free DNA and circulating tumor DNA, as well as their technical and clinical applications.

**Abstract:**

Current approaches for cancer detection and characterization are based on radiological procedures coupled with tissue biopsies, despite relevant limitations in terms of overall accuracy and feasibility, including relevant patients’ discomfort. Liquid biopsies enable the minimally invasive collection and analysis of circulating biomarkers released from cancer cells and stroma, representing therefore a promising candidate for the substitution or integration in the current standard of care. Despite the potential, the current clinical applications of liquid biopsies are limited to a few specific purposes. The lack of standardized procedures for the pre-analytical management of body fluids samples and the detection of circulating biomarkers is one of the main factors impacting the effective advancement in the applicability of liquid biopsies to clinical practice. The aim of this work, besides depicting current methods for samples collection, storage, quality check and biomarker extraction, is to review the current techniques aimed at analyzing one of the main circulating biomarkers assessed through liquid biopsy, namely cell-free nucleic acids, with particular regard to circulating tumor DNA (ctDNA). ctDNA current and potential applications are reviewed as well.

## 1. Introduction

**Figure 1:**
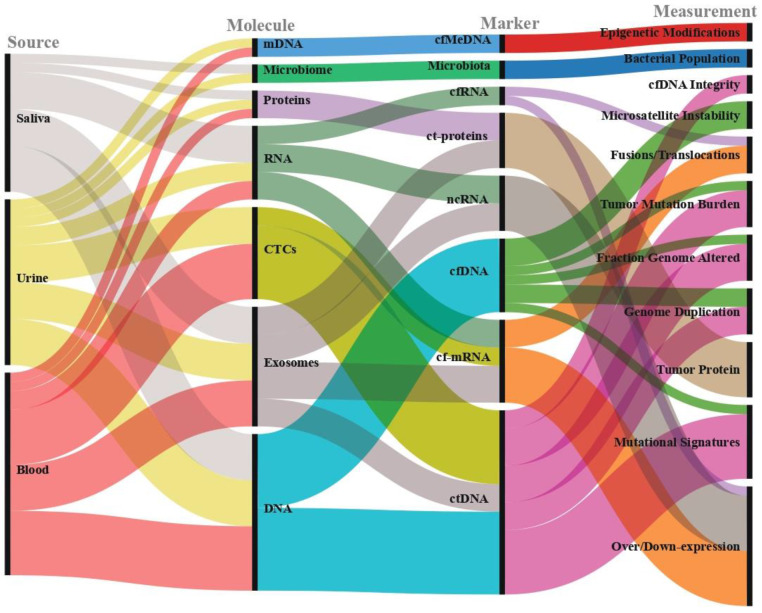
Visual representation of liquid biopsy applications through alluvial plot. The diagram flow shows the multitude of analytes (molecules) and applications (measurements) which can be assessed in liquid biopsy.

Over the last few years, the detection and utilization of circulating biomarkers for clinical purposes has emerged as a potential alternative and/or correlate to radiological procedures and traditional biopsies, especially in oncology. The detection of circulating cell-free nucleic acids (cfNAs) in plasma either in the form of DNA or RNA yields serious potential for clinical purposes in patients affected by cancer. Circulating tumor DNA (ctDNA), namely the fraction of cell-free DNA (cfDNA) in neoplastic patients derived from apoptosis and necrosis of tumor cells, or from processes of active release from neoplastic cells or extracellular vesicles, represents one of the most promising biomarkers, with serious potential for effective transition into clinical practice [1]. Other types of cfNAs include RNA-based biomarkers such as microRNA (miRNA) and non-coding RNA (ncRNA) [2]. As access to body fluids is less invasive, easier, faster and cheaper than performing a tissue/tumor biopsy, the analysis of circulating biomarkers obtained through liquid biopsies is gaining consideration for an effective transition to clinical practice. A comprehensive landscape of the possibilities of liquid biopsy molecular profiling is shown in Figure 1.

To date, the evaluation of ctDNA in oncology is still limited to specific clinical indications, such as the detection of actionable mutations in the bloodstream for a proper directioning of targeted therapy, while no actual indication is currently approved for cell-free RNA (cfRNA) [3]. An effective implementation of current workflows for cancer diagnosis and characterization by the assessment of cfNAs in the panorama of liquid biopsies would imply substantial progress toward a minimally invasive and personalized monitoring of neoplastic patients, possibly capturing the complex heterogeneity of the cancer mutational and transcriptional profile, without performing invasive procedures such as traditional tissue biopsies. One of the main challenges limiting the implementation of current clinical workflows with the assessment of these circulating biomarkers is the lack of standardized procedures for their detection, starting from pre-analytical elements such as sample collection and conservation, to cfNAs and isolation and analysis.

The main purpose of this review is to depict the current standards of pre-analytical and analytical processing of cfNAs. It is outside the scope of the present work to dissect in detail the current state of the art of their assessment in clinical practice, while we synthetically refer to ctDNA’s current and potential applications, alongside with cfRNA’s value for theranostic purposes, these being of particular interest for their immediate transferability potential in clinical practice.

## 2. Pre-Analytical Management of Samples

The integration of cfNAs assessment into clinical practice requires a proper standardization in order to guarantee reliable and reproducible results. To date, no shared consensus workflow in the assessment of cfNAs is available, as shown by the typical poor interlaboratory concordance [4,5,6,7]. Several studies have demonstrated the importance of evaluating aspects which influence the pre-analytical sample handling, such as the choice of collection tubes, use of stabilization reagents, storage conditions and temperature, sample processing, and extraction protocols. All these parameters can significantly affect quality and yield of cfDNA [8,9] and can lead to remarkable variability in cfDNA extraction efficacy, quantification and molecular characterization [10,11,12,13,14,15].

### 2.1. Sample Types

cfNAs are typically extracted from plasma or serum. Plasma is usually recommended for the extraction of cfDNA, due to serum contamination by genomic DNA (gDNA), mainly derived from blood cell lysis during coagulation [15]. cfNAs can be also assessed in other body fluids such as urine, saliva, tears, cerebrospinal fluid, alveolar lavage fluid, peritoneal fluid, seminal fluid, amniotic fluid and bone marrow, even though there is less evidence about their management and potential role for clinical purposes compared to blood samples. Moreover, the different biological conditions of diverse body fluids can affect the quantity and quality of extracted material. As an example, urine cell-free DNA (ucfDNA) undergoes degradation due to different factors such as glomerular filtration and the urinary environment, which cause higher DNA degradation compared to other body fluids such as serum and saliva [16]. The half-life of ucfDNA is therefore significantly shorter than plasma cfDNA. However, other biomarkers can be assessed in urine in addition to cfDNA, including circulating tumor cells, cfRNAs (miRNA, lncRNA and mRNA), proteins and peptides and exosomes [17]. Among the other body fluids, saliva presents the advantages of a non-invasive acquisition, avoiding the issues regarding personal privacy typically encountered in urine collection, but the procedures aimed at its collection are still far from a proper standardization [18].

### 2.2. Collection Tubes

The addition of anticoagulant solutions in blood collection tubes has a considerable influence on cfNAs. Different stabilizers can be used for this purpose, including EDTA and citrate. On the other hand, stabilization with heparin should be avoided since this molecule is an amplification inhibitor and interferes with PCR-based methods. Nonetheless, as confirmed by the literature, heparin immediately triggers the release of gDNA from leukocytes [19]. K2 or K3 EDTA represent the most common anticoagulants added to collection tubes used for cfNAs extraction. However, the use of K2/K3EDTA tubes requires a short time interval between sample collection and processing, typically <3 h at room temperature. A prolonged interval between sample collection and processing is typically associated with a significant increase in gDNA. Likewise, the limiting factors concerning the management of cfRNA include cfRNA degradation and the confounding background RNA derived from leukocyte lysis, which can preclude the detection of scarcely expressed targets [20]. Alternatively, different collection devices with diverse preservative reagents are available to prevent cell lysis (see Table 1 for a complete list of collection tubes specifically developed for the preservation of cfNAs obtained from different fluids). The main function of these tubes is the stabilization of white blood cells (WBCs), preventing the contamination of cfDNA with gDNA from lysed cells. This represents a capital prerequisite for the reproducibility of the analytical data. Blood, as well as other fluids, when treated with these stabilizing reagents, can be typically processed within 1 week from collection, with no noticeable alteration at polymerase chain reaction (PCR) amplification, whereas the use of formaldehyde or glutaraldehyde leads to a significant decrease in DNA amplification [21].

### 2.3. Centrifugation and Long-Term Storage

Centrifugation is necessary to remove all cellular components in excess from the blood, in order to obtain the plasma fraction harboring cfNAs. Hence, a correct procedure of centrifugation to decrease the contamination by gDNA is needed after whole-blood centrifugation (range 400–3000 rpm). Plasma is separated and collected with a pipette into a new tube, paying attention not to contaminate with the buffy coat. After this step, another centrifugation of the collected plasma is performed at high speed to remove the cellular debris. Centrifugation speed or temperature do not impact cfDNA recovery [35]. Page et al. demonstrated that a double centrifugation reduces the amount of longer, typically undesired, cfDNA fragments (>300 bp) [36]. Storage of processed plasma samples at −80 °C up to one year does not cause cfDNA degradation, while a longer storage may result in an up to 30% degradation rate per year [15,37,38]. Repeated cycles of freezing and thawing of whole blood and plasma are not recommended [15,37,38]. A protocol for the preparation of platelet-free plasma has been proposed as standard, showing particular efficacy in the analysis of microvesicles [39].

## 3. cfNAs Extraction

The monitoring and standardization of preanalytical conditions is a capital step for proper management of cfNAs extraction. Given the potential for an effective transition of liquid biopsies into clinical practice, relevant advancements can be observed in regard to the number and quality of extraction kits and methodologies, making in some cases the choice of the most appropriate kit rather difficult. A detailed list of the most common extraction kits for cf/ctDNA and cfRNA and their processing features is shown in Table S1. One of the main differences among extraction kits is the sample type they are suitable for. Most commercially available cfNAs extraction kits are compatible with either plasma or serum, and some of them have been adapted for extraction from urine or other body fluids and matrices such as saliva, cerebrospinal fluid, seminal fluid and stools. Nonetheless, there is a limited number of kits specifically designed for highly sensitive detection of cfNAs from urine. A further difference among different kits regards the underlying methods of cfNAs extraction. The two main techniques for cfNAs extraction rely on the use of magnetic beads or silica-membrane columns. In the first case, short DNA fragments bind selectively to magnetic particles that are subsequently detected through the use of a magnet; in the second case, fragments are adsorbed on a silica membrane surface and then purified through centrifugation or vacuum pressure. However, other methods have been developed by laboratories and companies, sometimes using unconventional reagents and systems not usually recommended for cfNAs extraction. A phenol-chloroform-based method and its subsequent modified version have been reported to efficiently isolate cfDNA from plasma [40], as well as the Triton/Heat/Phenol-based method [41]. Further uncommon approaches for the detection of cfNAs include the use of selective resins, such as the Wizard Resin/Guanidinium Thiocyanate and the Q Sepharose Anion Exchange Resin [42], or proprietary polymers, solid/liquid phase matrices and a filtration based method [42]. To date, however, the most innovative method for cfNAs detection (PIBEX) is based on microfluidics. PIBEX is a centrifugation-free extraction method which relies on the use of a silica membrane under vacuum pressure combined with an immiscible liquid, such as mineral oil [43]. This technology, validated for clinical purposes, uses a PIBEX chip, has the fastest extraction protocol available (only 15 min) and its performance in terms of cfDNA recovery rate does not show significant differences from the other common extraction kits [43]. Although most extraction kits are aimed only at research purposes, few are currently approved for clinical practice (CE-IVD), such as the QIAsymphony DSP Circulating DNA^®^ Kit (QIAGEN,** Hilden Düsseldorf, Germany) and the MagNA Pure 24^®^ Total NA Isolation Kit (Roche Diagnostics, GmbH, Mannheim, Germany). Moreover, some liquid biopsy diagnostic assays for tumor profiling include a dedicated cfDNA extraction method, providing an end-to-end workflow from extraction to analysis and reporting (AVENIO ctDNA Targeted Kit, Roche Diagnostics, GmbH, Mannheim, Germany. While in diagnostics, an automated extraction and handling of samples is preferred, as automation guarantees that the procedure is performed under standardized and controlled conditions, in the research setting manual procedures are more common. Using automated platforms has some advantages, such as the possibility of processing a higher number of samples in a single run (up to 96 in the QIAsymphony), and reducing hands-on time and total processing time [13]. Although it has been reported that the quality and quantity of cfDNA purified through automated platforms are comparable to those obtained through manual procedures [13], some works report that automated cfDNA extraction methods generally show lower recovery efficiency than manual methods [44]. When comparing extraction kits, data indicate the QIAamp Circulating Nucleic Acid Kit (QIAGEN, Hilden Düsseldorf, Germany) as the gold standard. This kit demonstrated superior recovery efficiency over various other methods in numerous comparative studies [10,45,46]. However, other commercial kits were found to produce yields of cfNAs comparable to QIAamp [12,14,46]. In recent years, many laboratories have been implementing the use of magnetic beads-based extraction, for both its cost-effectiveness and rapidity of execution. The QIAamp MinElute cfDNA Kits (QIAGEN, Hilden Düsseldorf, Germany) and the QIAsymphony (QIAGEN, Hilden Düsseldorf, Germany) have been shown to recover the greatest amount of cfDNA from plasma, obtaining a relevant concentration of cfDNA in the eluate, especially in regard to cfDNA short fragments (50–250 bp) [44,45]. As fluids collected through liquid biopsies also include miRNA, exosomes and vesicular nucleic acids, suitable kits are required for their extraction. Only a small number of the kits listed in Table 2 are able to separate miRNA and vesicular NAs, in addition to cfDNA/RNA extraction. Examples of efficient and functional miRNA extraction kits are the PureLink miRNA Isolation Kit (Thermo Fisher Scientific, Waltham, MA, USA), the Maxwell RSC miRNA Plasma and Serum Kit (Promega, Madison, WI, USA) (also suitable for previously isolated exosomes), the mirPremier^®^ microRNA Isolation Kit (Merck, Darmstadt, Germany), the mirVana miRNA Isolation Kit (Ambion, Thermo Fisher Scientific, Waltham, MA, USA) and microRNA purification Kit (Norgen Biotek, Corp., Thorold, ON, Canada).

## 4. cfNAs Quality Control

Traditionally, fluorescence-based assays, such as PicoGreen^®^ and Qubit^®^ (Invitrogen, Thermo Fisher Scientific, Waltham, MA, USA), have represented the standard for the assessment of cfNAs concentration. Moreover, quantitative PCR (qPCR) is among the most widely used methods for cfNAs quantification as well [47]. These techniques were widely used for their cost-effectiveness, rapidity of execution, and reproducibility but show, however, some limitations; only providing information about the total yield of cfDNA, without characterizing cfDNA subcomponents or detecting the possible contamination by high molecular weight (HMW) DNA [48]. The presence of HMW DNA in cfDNA samples can negatively affect sequencing quality. The Agilent Cell-free DNA ScreenTape is able to provide reliable total DNA concentration as well as quantitative assessment of cfDNA subcomponents apart from HMW DNA. The assay also provides the percentage of cfDNA subcomponents. In addition, only 2 μL of cfDNA from each sample is required for this test. Furthermore, the separation of cfDNA on the Agilent Femto Pulse system with the Agilent Ultra Sensitivity NGS kit (Agilent Technologies, Santa Clara, CA, USA) allows the assessment of sizing, quantification and resolution of cfDNA fragments even at low picogram concentrations. The electrophoretic profiles displayed by the Agilent Cell-free DNA ScreenTape include marks for mono-, di- and tri-nucleosomal DNA fragments, respectively, at 170, 350 and 550–580 bp. At the same time, by using methods based on capillary electrophoresis, it is possible to estimate miRNA concentration, before expression analysis. Moreover, the libraries generated for NGS technologies are usually quantified by qPCR with a Kapa Quantification Kit (Roche Diagnostics, GmbH, Mannheim, Germany) and in a Quantifluor (Quantus Fluorometer, Promega, Madison, WI, USA), using a QuantiFluor ONE dsDNA Kit (Promega, Madison, WI, USA). Library size is generally checked on the Bioanalyzer (Agilent Technologies, Santa Clara, CA, USA) using high-sensitivity DNA chips (Agilent Technologies, Santa Clara, CA, USA) or HS DNA ScreenTape on Tapestation 2200/4200.

## 5. cfNAs Technical Applications

### 5.1. Mutation Detecation

The detection of mutations in cfNAs is one of the main applications of liquid biopsies. The analysis of somatic mutations in cfNAs extracted from diverse body fluids is fundamental for detecting ctDNA released from tumor cells, which corresponds to a small percentage of the total cfDNA [49]. The extreme dilution of ctDNA in cfDNA, especially in early-stage cancers, is one of the main difficulties encountered in its detection. Moreover, the selection of fragments of appropriate length can be difficult as well [50]. For all these reasons, standard sequencing approaches (Sanger sequencing, pyrosequencing) are not adequate to investigate the mutational status of ctDNA. As these approaches have some technological limitations in terms of sensitivity, they may be employed exclusively in cases with high tumor burden and high levels of ctDNA. Currently, one of the most common approaches for mutation detection in body fluids is single gene testing performed by PCR technology in the form of conventional PCR-based methods, such as qPCR, amplification refractory mutation system PCR (ARMS-PCR), co-amplification at lower denaturation temperature-PCR (COLD-PCR), bidirectional pyrophosphorolysis-activated activated polymerization (bi-PAP), MIDI-activated pyrophosphorolysis (MAP) and digital PCR-based methods, such as digital droplet PCR (ddPCR) and BEAMing (Beads, Emulsion, Amplification, Magnetics) [51]. Of note, the detection of known mutations in ctDNA is also accomplished through PCR-based methods that use unconventional molecular biology techniques, such as allele-specific, non-extendable primer blocker PCR (AS-NEPB-PC) or PNA/LNA-PCR, which incorporates locked or peptide nucleic acid residues. PCR-based methods generally require small sample input volumes, and have relatively low costs and short turnaround times; on the other hand, they do not represent the most specific and sensitive approach for mutation detection. However, the latest digital PCR technologies, based on the combination of microfluidics and emulsion PCR to generate sized droplets [52], are able to identify low-allelic-frequency alterations with sensitivity up to 0.01% for BEAMing and between 0.05 and 0.001% for ddPCR [53]. Given its high sensitivity, digital PCR shows some unquestionable advantages: for instance, it allows the detection of as little as one targeted mutation on ctDNA, thus distinguishing ctDNA from non-tumor cfDNA. Furthermore, this technique is able to quantify and identify mutated copies from wild-type copies [54,55]. As the main limit of PCR-based methods is that they have very limited multiplex capacity and can investigate only a restricted number of genes, NGS approaches have rapidly entered this field and have been implemented for mutation detection on ctDNA. These high-throughput procedures do not show a deeper sensitivity but give the possibility to simultaneously detect a high number of molecular aberrations such as single nucleotide variants (SNVs), insertions and deletions, through massive parallel sequencing [56]. NGS-based assays, using PCR amplicons or hybrid capture probes, have achieved promising results in the detection of new mutations, performing accurate and reproducible analysis [57]. Comprehensive genomic profiling, namely whole-genome sequencing (WGS) and whole-exome sequencing (WES), have proven to be feasible on ctDNA [58]. However, these approaches show some limitations, such as high costs, long turnaround times and large quantities of sample input. In addition, WES also shows the issue of a relatively low limit of detection and the risk of false positives due to artefacts. For all these reasons, WGS and WES have been rapidly replaced by targeted sequencing, which investigates only a subset of cancer-related genes contained inside a panel. Although some gene panels commonly used for genotyping DNA extracted from fresh frozen or formalin-fixed paraffin-embedded tissue samples are applied to liquid biopsy, there are many targeted panels specifically designed for ctDNA detection, such as FoundationOne Liquid CDx (Foundation Medicine, Cambridge, MA, USA), TruSight Oncology500 ctDNA Assay (Illumina, San Diego, CA, USA), Tempus xF liquid biopsy assay (Tempus, Chicago, IL, USA), AVENIO ctDNA Targeted Kit (Roche Diagnostics, GmbH, Mannheim, Germany), Archer Reveal ctDNA 28 Kit (Archer Diagnostics, Boulder, CO, USA), Guardant360 cfDNA assay (Guardant Health, Redwood City, CA, USA), PlasmaSELECT 64 (Personal Genome Diagnostics, Baltimore, MD, USA) and others. Their analytical and clinical validity have been assessed by many works [59,60,61]. As these panels are hardly ever customizable, some companies and institutions have decided to design and test their own gene panels. These in-house developed panels, that generally include a smaller number of genes, could enter clinical practice as soon as they are clinically validated, such as Target Selector ctDNA assay [29,62,63]. Currently, ultrasensitive deep targeted sequencing is the technology of choice for mutation detection on ctDNA [64]. The use of unique molecular identifiers (UMIs or UIDs), applied to this type of sequencing, has dramatically improved the results from such technologies. UMIs and UIDs are molecular barcodes, usually short random nucleotide sequences, that are ligated to amplicons or hybridized sequences during library preparation, allowing for a reduction in background signals, and correcting both DNA polymerase-induced errors and uneven amplification when used in combination with deep sequencing [65,66]. There is a wide range of NGS-based technologies performing targeted sequencing through the use of gene panels aimed at mutation detection. Examples include TAm-Seq (Tagged-amplicon deep sequencing) [67], Safe-SeqS (Safe-Sequencing System) [68], SiMSen-Seq (Simple, Multiplexed, PCR-based barcoding of DNA for Sensitive mutation detection using Sequencing) [69], CypherSeq [70], DuplexSeq [71], smMIPs (Single molecule Molecular Inversion Probes sequencing) [72], BAsE-Seq (Barcode-directed Assembly for Extra-long Sequences) [73] and CAPP-Seq (Cancer Personalized Profiling by deep sequencing) [74]. Although these assays are different in terms of ctDNA input required, they are all UMI-based methods, which combine library preparation, UMI tagging, sequencing and statistically-based analysis algorithms with a detection limit ranking from 0.1 (Safe-SeqS) to 0.004% (CAPP-Seq) [47,75]. However, an associated limit of such low sensitivities is the actual absence of so much cf/ctDNA in plasma. So far, CAPP-Seq appears to be the most specific and sensitive NGS-based technology and its use is increasingly being implemented in clinical practice. Its main advantage is the possibility of detecting extremely low concentrations of mutations from small ctDNA inputs. In addition, it uses “selectors” consisting of biotinylated DNA oligonucleotides that are complementary to recurrently mutated regions in the cancer of interest [74]. Another upfront approach for mutation detection on ctDNA is single-molecule sequencing. This method is performed through nanopore sequencing and is mainly represented by INC-Seq (Intramolecular-ligated Nanopore Consensus Sequencing). This sequencing technology is completely different from canonical sequencing as it uses rolling circle amplification (RCA) of circularized templates to generate linear products that can be sequenced on the nanopore platform [76]. Additionally, another valuable approach for single-molecule sequencing is given by PacBio technology (Pacific BioSciences of California, Inc, Menlo Park, CA, USA). Besides PCR and NGS, other systems for mutation detection include matrix-assisted laser desorption/ionization (MALDI), surface-enhanced Raman spectroscopy (SERS), electrochemical chips and fluorescently coded microparticles [77]. In particular, UltraSEEK, which uses a mass spectrometry-based approach for high-throughput, multiplexed, ultrasensitive mutation detection, is gaining more and more interest, as it allows saving time and biological material without compromising analytical sensitivity and accuracy [78]. Finally, all these methods for mutation detection on ctDNA benefit from recent advances in downstream data analysis, with the elaboration of NGS data aimed at detecting DNA variants at extremely low frequency. A promising example of it is ERASE-Seq (Elimination of Recurrent Artifacts and Stochastic Errors Sequencing). This highly accurate and sensitive technology is able to significantly decrease false positives when the analysis is performed thanks to the use of a solid statistical framework in combination with efficient error modeling [79]. A summary of ctNAs’ technical applications, including NGS-based analysis, is provided in Table 2.

### 5.2. Other Genetic Alterations

Besides mutation analysis, ctDNA is being increasingly studied for investigating other genetic alterations, using NGS as the technology of choice. Currently, cancer-specific copy number variations (CNVs) can be analyzed by performing low-pass sequencing on ctDNA followed by normalization algorithms [80]. Although it has low coverage (0.1–0.5×) and requires a ctDNA fraction above 5% to achieve good specificity and sensitivity, shallowNGS allows the detection of gene amplifications or deletions using an average ctDNA input of 10 ng [81,82]. However, some of the most recent approaches of ultrasensitive deep targeted sequencing are often applied to CNVs detection in combination with mutation analysis, such as TAm-Seq and CAPP-Seq. Conversely, other NGS-based methods are specifically intended for CNVs identification. An example of such methods is Fast-SeqS (Fast Aneuploidy Screening Test Sequencing System). Although it was initially developed for the prenatal screening of fetal chromosomal status, it has been subsequently implemented for the analysis of ctDNA samples derived from cancer patients. Through this efficient technology, around 38,000 amplicons are amplified using only one primer pair. During the amplification step, degenerate bases at the 5′-end of the primer are used as molecular barcodes to label each DNA template, ensuring that each molecule is counted only once [83]. A modified version of this method (mFAST-SeqS) has been reported to estimate the amount of ctDNA in plasma in a cost-effective and rapid manner without any prior knowledge of specific aberrations of the primary tumor [84]. In recent years, two other approaches, namely massively multiplexed PCR and next-generation sequencing (mmPCR-NGS) and Repetitive Element AneupLoidy Sequencing System (RealSeqS), have been proven to accurately detect CNVs in liquid biopsy. mmPCR-NGS can identify both clonal and subclonal CNVs with average allelic imbalances as low as 0.5% and has already been tested on plasma samples and matched tumor tissue subsections of different cancer types in order to assess its analytical sensitivity [85]. On the other hand, RealSeqS appears to be the most powerful evolution of FAST-SeqS, as it allows for the detection of somatic mutations and CNVs, as well as focal amplifications and deletions, all requiring as little as 0.1–0.25 ng of ctDNA [86]. With regard to other genomic alterations, ctDNA assessment can be applied for the detection of fusion genes. Many validated plasma-based multigene assays such as Guardant360 include fusion genes in their panels; thus, diverse and potentially actionable fusions can be detected using ctDNA assays [87,88]. Although there is enough evidence over the past 1–2 years supporting that plasma genotyping using hybrid-capture NGS technology can reliably detect fusions, such as ALK or ROS fusions in NSCLC patients [89], this analysis is not routinely performed on ctDNA. A more promising application of ctDNA is the estimation of tumor mutational burden (TMB) and microsatellite instability (MSI), which are generally assessed through WES and NGS hybrid-capture methods in liquid biopsy. TMB, defined as the number of non-synonymous mutations per megabase in a neoplastic specimen, is mainly referred to as blood TMB (bTMB) when dealing with ctDNA. The bTMB assay was first developed by Foundation Medicine. This assay detects somatic base substitutions down to 0.5% allele frequency across 394 genes from as little as 1% tumor content in a cell-free DNA sample. The main difference between tissue TMB and bTMB is that the latter analyzes only SNVs, whereas tissue TMB also includes analysis of indels and fusions. However, bTMB appears to effectively correspond to TMB, and it has been already validated in clinical practice for certain cancer types [90,91]. Similarly to bTMB, MSI detection on ctDNA is performed through targeted sequencing. The ctDNA-based MSI detection using Guardant360 was found to be highly concordant with tissue-based testing and represents an analytically and clinically validated assay in this field [92].

### 5.3. The Cell-Free RNA Transcriptome

Concerning RNA-based non-invasive biomarkers, non-coding RNAs including miRNA and lncRNA have been studied extensively in multiple diseases [2]. Quantitative reverse transcription PCR (qRT-PCR) assays and microarrays are frequently used for the quantification of both miRNAs and lncRNAs, although these techniques are only able to investigate predefined targets [93,94]. FireFly particle technology uses an interesting approach that enables the detection and quantification of miRNAs by flow cytometry [95]. Particles contain three distinct functional regions, each separated from the other by inert spacer regions. The central analyte quantification region contains probes that capture target miRNAs. The two end regions function as parts of a barcode; the included software uses this barcode to identify which target miRNA species has been captured by the particle. These multiplex assays allow for the simultaneous measurement of up to 68 target miRNAs. The application of the FireFly methodology allowed assessment of the enrichment of miR-200 family miRNAs in extracellular vesicles (EVs) from metastatic breast cancer cell lines, which was found to correlate with the metastatic potential of metastatic tumors in mice [43]. Whole-transcriptome RNA sequencing represents the main approach for exploratory aims, although the interpretation of NGS data requires sophisticated bioinformatics analysis [93]. The amount of input material required for NGS library preparation widely varies for short ncRNAs and lncRNAs. Existing technologies are able to prepare short miRNA libraries using up to 1 ng of input material [96,97]. Although multiple RNA-seq modifications for lncRNA analysis have been reported [98], most are not applicable to liquid biopsy assays for the high amount of input material required (>1 μg). Furthermore, all of the aforementioned techniques require cDNA generation and PCR-based (pre)amplification steps, and the efficiency of reverse transcription was shown to depend on the enzyme used, as well as on RNA integrity and concentration [99].

Concerning coding RNAs, the cell-free messenger RNA (cf-mRNA) transcriptome can be considered as a compendium of transcripts collected from all organs [100]. NGS-based whole transcriptomic profiling of cf-mRNA was conducted by Ibarra et al. in order to understand the biological origins of cf-mRNA: this study strongly suggested that living cells release cf-mRNA into circulation, displaying the potential of circulating transcripts as non-invasive and informative biomarkers [101].

### 5.4. Technologies for cfDNA Methylation Assessment

cfDNA methylation is a promising and informative biomarker in cancer diagnosis, prognosis and prediction of therapeutic response. As the cfDNA amount in plasma is low, methods for the quantification of cfDNA methylation need to be highly sensitive and specific. Another issue concerning cfDNA is its high degradation and possible contamination with gDNA. Recently, different approaches for the measurement of cfDNA methylation have been proposed.

The most studied epigenetic marker in cancer is 5-methylcytosine (5mC), even though also its hydroxylated analog (5-hydroxymethylcytosine (5hmC)) presents serious potential as an epigenetic marker for cfDNA analysis [102]. Currently, a few low-throughput techniques such as nanopore and single-molecule sequencing assess a direct detection of methylated DNA (MeDNA) [103]. The most used methods for DNA preprocessing enable the use of techniques such as PCR, microarray and sequencing and comprise restriction enzymes (MREs) digestion, bisulfite treatment, affinity enrichment or combinations between enzymatic and chemical modification proceeding. MREs are traditionally applied for methylation analysis. Two kinds of enzymes are used: methylation-sensitive enzymes that cut only unmethylated DNA, leaving the methylated DNA intact, or methylation-insensitive enzymes. MREs digestion can be followed by PCR [104], microarray [105] or sequencing [106]-based assays. Despite its cost effectiveness and reproducibility, DNA digestion by MREs is an error-prone method, and provides information about the enzyme-specific CpG sites only, with limited applicability to the typically highly fragmented cfDNA samples.

The gold standard for the detection of MeDNA is sodium bisulfite treatment, which permits its quantitative and qualitative assessment at single base pair sensitivity [104]. This technique is able to convert every unmethylated cytosine to an uracil residue, which subsequently changes into a thymine nucleotide when the sense strand is amplified. By contrast, the cytosine nucleotides found on the amplified sense strand represent the 5-methylcytosine (5mC) residues which are not affected during sodium bisulfite treatment. This method is relatively cheap, feasible and rapid. The bisulfite treatment, however, causes random DNA breaks, as well as DNA degradation caused by critical chemical conditions including low pH values, high temperatures, long incubation times, high concentration of bisulfite and alkali treatment [107]. Another possible disadvantage of this method correlated with low DNA quantity and quality is the partial conversion efficiency and non-specific conversion of 5mC also caused by chemical conditions, purification procedures and the possible presence of conversion-resistant sequences, which can produce false-positive or false-negative rates [108]. In addition, this method cannot distinguish 5mC from 5hmC, as both are unable to be converted to uracil [109]. There are several commercial bisulfite conversion kits available to DNA from body fluids [110].

Other methods frequently used to evaluate DNA methylation status include methylation-specific PCR (MSP) [111]. In this method, DNA, after bisulfite treatment, undergoes PCR with two primer pairs. The first primer pair recognizes and anneals only to the methylated DNA region, while the second primer pair amplifies unmethylated DNA sequences. This method is highly sensitive and used for diagnostic purposes, as MSP reactions are able to detect a single methylated allele among around one thousand unmethylated ones [112]. Unfortunately, MSP shows some disadvantages, such as its limited use only for quantitative analysis. However, MSP has been implemented with several real-time PCR adaptations. The quantitative MSP (qMSP) is highly specific and more sensitive compared to conventional PCR and thus represents an appropriate method for cfDNA methylation analysis [113]. Methylation-specific qPCR (e.g., EpiTect MethyLight PCR Kit, QIAGEN, Hilden Düsseldorf, Germany) combines conventional MSP with a TaqMan probe. Depending on the methylation status of the targeted sequence, DNA is hybridized alternatively by the TaqMan^®^ probe specific for bisulfite-treated methylated DNA or the TaqMan^®^ probe specific for unmethylated DNA. Both probes are labeled with different fluorophores, which are released during PCR at the moment of hybridization to the DNA. Fluorescence is proportional to the amount of accumulated PCR product [114]. This technique is relatively expensive compared to other SYBR Green-based qMSP methods.

Another valuable method to enrich MeDNA is given by affinity enrichment techniques through methyl-binding domain (MBD) proteins or 5mC-specific antibodies (MeDIP). In MeDIP, the DNA sequence containing a targeted 5mC is immunoprecipitated with monoclonal antibodies after denaturation [115]. PCR, array and sequencing-based techniques are all appropriate tools to analyze immunoprecipitated DNA with a resolution of 100 bp [116]. In order to apply MeDIP-seq to methylation analysis of low-input cfDNA, a novel protocol was developed combining cell-free methylated DNA immunoprecipitation and high-throughput sequencing (cfMeDIP-seq). To increase the initial DNA input, this method uses exogenous lambda DNA as a filler [117]. The use of filler DNA can reduce the input of cfDNA to 1–10 ng. The filler DNA guarantees an efficient immunoprecipitation between samples with different cfDNA input, because it maintains a constant antibody/DNA ratio and minimizes non-specific binding and DNA loss [118]. The advantage of using lambda DNA is that it has no sequencing adapters, hence it does not undergo a subsequent amplification and does not interfere with the analysis of sequenced data.

MethylCap is a method which captures MeDNA with the MBD domain of MeCP. A DNA fragment is captured by a recombinant protein GST–MBD resulting from the fusion of MBD and glutathione-S-transferase protein (GST) and by paramagnetic beads. This technology, performing under low-salt conditions, allows the stratification of DNA fragments according to the level of methylated CpG density, and their subsequent sequencing [119]. Both MethylCap and MeDIP methods are able to detect only 5mC. While MethylCap binds double-stranded DNA, does not need the denaturation step and is able to capture fragments with higher CpG density, MeDIP binds single-stranded DNA to capture methylated DNA, and is suitable to select methylated regions with low CpG density better than MethylCap [120].

Recently, enzymatic methyl-seq (EM-Seq) and ten-eleven translocation (TET)-assisted pyridine borane sequencing (TAPS) have been developed. These two methods combine the use of enzymatic and chemical modifications. EM-Seq is based on two sets of enzymatic reactions. During the first step, two enzymes, TET2 and T4-bGT, convert 5mC and 5hmC into substrates that will not be deaminated by APOBEC3A. In the second step, APOBEC3A deaminates only unmodified cytosines, and converts them to uracils. The preservation of 5mC and 5hmC allows the discrimination of cytosines from 5mC and 5hmC [121]. Converted sequences are identical to those obtained after bisulfite treatment and can be analyzed with the same downstream techniques. TAPS uses a combination of TET oxidation of 5mC and 5hmC to 5-carboxylcytosine (5caC) with pyridine borane reduction of 5caC to dihydrouracil (DHU). Subsequently, PCR converts DHU to thymine, inducing a C-to-T transition of 5mC and 5hmC [122]. In contrast to bisulfite conversion, this reaction requires double-stranded DNA and as DNA integrity is preserved it is possible to use low-input amounts (TAPS 1 ng cfDNA; EM-seq 100 pg) to generate high-quality sequencing data [123]. EM-Seq has been used to create cfNOMe (cell-free DNA-based nucleosome occupancy and methylation profiling); this technique permits the evaluation of nucleosome position and methylation status with a single assay on cfDNA from biopsy liquid [124].

## 6. Current Challenges in ctDNA Detection and Strategies for Its Optimization

As outlined in the introduction, various challenges currently limit the applicability of liquid biopsies and ctDNA to clinical practice. ctDNA concentration in blood and other body fluids, such as urine, saliva and cerebrospinal fluid, depends on factors such as tumor burden, vascularization, cellular turnover and anatomical site [125].

The correlation between ctDNA fraction in cfDNA and tumor stage is widely recognized, with the former possibly representing up to 10% of the latter in late-stage cancers [125]. Consistently, ctDNA in early stages or resected cancers accounts for a restricted portion of cfDNA, implying significant difficulties in its detection.

All molecular aberrations occurring in the DNA of cancer cells are present and possibly detectable in ctDNA. These include somatic and germinal mutations, microsatellite instability, loss of heterozygosity, alterations in methylation, and copy number variations [126,127,128]. When coming to ctDNA evaluation for clinical purposes, however, several difficulties are encountered.

The genomic approach applied for ctDNA detection, based on sequencing somatic mutations suggestive of carcinogenic modifications from cfDNA, in fact lacks both sensitivity and specificity. The former issue mainly depends on the restricted percentage of ctDNA in cfDNA in non late-stage cancers, together with the low rate of recurrent pathogenic mutations occurring in ctDNA, while the latter is to be attributed to non-specific mutational profiles occurring in cfDNA of healthy individuals [117,129]. Clonal hematopoiesis is one of the main confounding factors altering the mutational profile of cfDNA in healthy individuals. This phenomenon, probably correlated with cells aging, refers to the presence of clonal populations of myeloid cells in the bloodstream harboring somatic mutations often in genes of interest for the detection of ctDNA, such as *JAK2, TP53, BRAF, KRAS* and *PIK3CA* [3].

Diverse strategies have been developed in order to overcome the interference of confounding clonal phenomena. The use of paired ddPCR or NGS-based techniques on both the primary tumor and plasma allows the selection of mutations previously detected in the primary site through the use of personalized assays, even though this approach is applicable only after tumor diagnosis and characterization through traditional tissue biopsy [3]. Moreover, the release of highly fragmented ctDNA fragments in the bloodstream impacts cfDNA integrity, depending on tumor burden. ctDNA fragments, typically sized between ~130 and ~170 bp, with a peak around 166 bp, seem in particular to derive from apoptotic processes of cell death [125,130]. However, the association between cfDNA integrity and patients’ prognosis is still unclear, as an increase in the former has been reported either as a good and a poor prognostic factor [131]. Approaches combining the assessment of cfDNA fragmentation and sequencing, however, have been reported to augment the accuracy of ctDNA detection, as assessed by Mouliere et al. and Cristiano et al. [130,132].

Finally, ctDNA assessment can be combined with several other biomarkers or techniques for an overall improvement in performance. The application of so-called “multi-omics”, i.e., the joint detection of different biomarkers or the use of multiple methods with different underlying principles for clinical purposes, has already shown a significant improvement compared to the assessment of individual biomarkers, especially in the setting of early-stage disease [133]. The conjunct assessment of different biomarkers can be performed with different levels of integration: elementary integration can be referred to the evaluation of biomarkers of the same type, such as DNA–DNA combinations, while advanced integration may involve the contemporary assessment of different kinds of biomarkers, such as proteins and circulating DNA, or the conjugation of liquid biopsies with radiological procedures [133].

## 7. Current and Potential Applications of ctDNA Assessment

### 7.1. Assessment of Actionable Mutations for Therapeutic Purposes and Detection of Primary and Secondary Resistance to Systemic Therapy

The minimal invasiveness of ctDNA assessment, together with the possibility of capturing and monitoring over time the genomic heterogeneity of the cancer mutational landscape, makes this kind of procedure of particular interest for the selection of the most appropriate drug, in the paradigm of targeted therapy. To date, the research of actionable mutations in plasma cfDNA for the administration of targeted therapy is indeed the only clinical application approved by the FDA in oncology in regard to ctDNA assessment.

PCR-based assays typically yield high specificity (>90%) and suboptimal sensitivity (<70%) in detecting mutations assessed in the primary tumor, even though the latter can be increased by using ddPCR [3]. This implies that the identification of somatic mutations in cfDNA, previously assessed through tumor tissue assays, can effectively direct the administration of targeted therapy, while the inability of detecting actionable mutations in cfDNA may imply the execution of further tissue assays. The first test approved by the FDA (cobas EGFR mutation test v2) was aimed at screening patients affected by advanced non-small cell lung cancer (NSCLC) for circulating EGFR mutations, in order to assess their eligibility for erlotinib, an EGFR tyrosine kinase inhibitor [15]. Therascreen PIK3CA RGQ PCR kit, another PCR-based assay, was approved for the assessment of PIK3CA mutations in plasmatic cfDNA collected from patients affected by advanced hormone receptor-positive HER2-negative breast cancer. The effective detection of PIK3CA mutations implies the eligibility for the combination of alpelisib, a PIK3CA inhibitor, and fulvestrant [134,135].

The first FDA-approved multi-gene testing (Guardiant360 CDx assay) on cfDNA, which aims at detecting EGFR mutations in patients affected by NSCLC who can benefit from the use of osimertinib, dates to August 2020 [136]. This assay allows the assessment of SNVs in 55 cancer-related genes. F1 Liquid CDx, approved in October 2020, is another example of an assay aimed at comprehensive genome profiling for therapy directioning in NSCLC, prostate cancer, ovarian cancer and breast cancer [137]. Its current indications include the identification of BRCA1 and BRCA2 mutations in patients affected by ovarian cancer potentially eligible for rucaparib, the detection of ALK rearrangements in patients affected by NSCLC potentially eligible for alectinib, the detection of PIK3CA mutations in patients affected by breast cancer potentially eligible for alpelisib and the detection of BRCA1, BRCA2 and ATM mutations in patients affected by castration-resistant prostate cancer who can beneficiate from treatment with olaparib.

Furthermore, the assessment of cfDNA mutations underlying primary resistance or the subsequent occurrence of resistance mechanisms to targeted therapies can be exploited for a proper administration or update of systemic treatment, avoiding exposing patients to the often considerable side effects of ineffective agents given tumor primary or secondary resistance possibly occurring after clonal expansion of resistant tumor cells. Several studies report the potential clinical utility of ctDNA monitoring during systemic treatment for these purposes. The detection of the EGFR T790M mutation has been reported as associated with resistance to EGFR tyrosine kinase inhibitor in NSCLC [138], while the role of circulating RAS mutations assessment in plasma has been depicted for colorectal cancer and melanoma for both the prediction of response to therapy and early detection of resistance [139,140]. The detection of several mutations in ctDNA of breast cancer patients, such as PIK3CA, TP53 and TERT, was reported as associated with resistance to therapy and disease progression [141]. Another explicative example concerning breast cancer is given by the emerging role of ESR1, the gene encoding ERα, part of the estrogen receptor. ESR1 mutations, besides being associated with shorter overall survival and progression-free-survival, are a cause of endocrine therapy failure, occurring typically in the metastatic setting [142].

However the detection of specific circulating mutations for cancer profiling is not the only way to exploit ctDNA for theranostic purposes. TMB has indeed shown concrete value as a predictor of response to immune checkpoint drugs for diverse types of cancer, it being directly proportional with the generation of neoantigens recognized by the immune system [143]. In addition, the correlation between TMB and the impairment of DNA damage repair mechanisms may imply its possible application for the prediction of the response to chemotherapy or radiotherapy, even though this option still has to be carefully evaluated. At the same time, the repeated quantitative assessment of ctDNA can be exploited for the monitoring of the response to systemic treatment, with its reduction typically associated with radiologically-proven response to therapy and its increase correlated with tumor progression and shorter disease-free survival or progression-free survival (DFS), possibly implementing standard imaging aimed at patients’ staging or detection of relapse during systemic treatment [144,145,146].

### 7.2. Early Diagnosis

To date, no effective protocol for ctDNA detection for diagnostic purposes yields sufficient clinical validity for effective approval into clinical practice. The detection of somatic mutations from cfDNA either via targeted sequencing or WGS or WES presents relevant limitations in terms of both sensitivity and specificity, as outlined above.

At the same time, however, approaches based on the epigenomic features of cfDNA may potentially overcome these issues. DNA methylation is consistent with cellular and tissue origin [147], making cfMeDNA a promising biomarker for non-invasive assessment of solid tumors. Furthermore, methylation changes play an important role in different kinds of cancers, occurring in the early phase of carcinogenesis, usually undermining the expression of tumor suppressor genes [148].

Approaches based on bisulfite treatment, with the conversion of unmethylated cytosine residues to uracil, traditionally applied for the assessment of cfMeDNA, are inefficient due to the degradation of input DNA. Results from the Circulating Cell-free Genome Atlas Study (CCGA) concerning the assessment of methylation signatures in cell-free DNA, possibly the largest study investigating methylomic features for diagnostic purposes in neoplastic patients, show inadequate accuracy in the detection of early-stage cancers through plasma cfDNA bisulfite sequencing, with sensitivity for the detection of stage I and II cancers of <20 and <50%, respectively [149].

However, new immunoprecipitation-based methods for the assessment of cfMeDNA seem to overcome the limitations of bisulfite sequencing. The protocol elaborated by Shen et al., through the enrichment of CpG-rich, potentially more informative fragments, requires in fact <10 ng of input DNA, with effective implications for both clinical applicability and cost-effectiveness. This protocol has been independently validated for renal cell carcinoma and intracranial tumors, with promising results either in early- or late-stage neoplasms [150,151]. Of interest, cfMeDIP-seq for the detection of renal cell carcinoma has been performed on both plasma cfDNA and urinary cfDNA (ucfDNA), achieving an overall accuracy of 0.99 and 0.86 across all stages, respectively, even without being specifically designed for ucfDNA evaluation. The assessment of clinical validity of ucfDNA for diagnostic procedures is of great interest, as its validation would represent an important advancement for the management of large-scale screening protocols.

A methylome-based assay (Panseer) showed also promising results in the early diagnosis of gastric, esophageal, colorectal, lung and liver cancer, managing to detect these neoplasms in asymptomatic individuals up to four years before standard diagnostic procedures [152]. Even in cases of validation, the clinical implications of these results are debatable. In particular, does the detection of these methylomic signatures subtend a high-risk condition which degenerates in cancer in the majority of patients, or does it subtend an occult ongoing carcinogenic process which will be clinically manifested only after years? In addition, does the detection of a methylomics signature suggestive of cancer justify early treatment in asymptomatic individuals? These issues should be carefully addressed in order to possibly insert the assessment of tumor cfMeDNA into current clinical workflows.

### 7.3. Early Detection of Recurrence/Relapse

Early detection of cancer relapse is particularly desirable in a context of increased rates of long-term survival among neoplastic patients. ctDNA detection subtends the persistence of clinically occult minimal residual disease (MRD) after surgery or systemic treatment, either in the adjuvant or neoadjuvant setting, and therefore it is generally regarded as a poor prognostic factor, as well as a predictor of local or metastatic recurrence. On the contrary, its complete clearance is typically associated with a prolonged DFS [153,154,155]. A common approach to the early detection of local or distant relapse is the research via ddPCR of somatic mutations in plasma, previously identified through sequencing of primary tumor DNA, with the construction of personalized tumor-specific assays for the assessment of ctDNA. This approach yields serious potential in breast cancer, with ctDNA detection reported as having a lead time of over 10 months compared to clinical relapse, possibly enabling the categorization of patients by the risk of relapse [156]. In this scenario, ctDNA detection would be particularly useful as an effective surrogate for MRD assessment in patients affected by neoplasms presenting high chances of relapse, such as lung cancer; an early detection of ctDNA harboring actionable mutations would allow the early treatment of relapse, possibly enhancing the efficacy of chemotherapy by administering targeted agents [157]. The possibility of MDR detection through ctDNA assessment should be carefully evaluated, being of interest for the potential complete eradication of the tumor or, at least, the postponement of clinically evident relapse or progression to metastatic disease.

### 7.4. ctDNA as An Independent Prognostic Factor

Being directly correlated with cellular proliferation and tumor vascularization, cfDNA and ctDNA levels are recognized as independent surrogates of tumor burden. cfDNA quantity in the bloodstream of neoplastic patients is influenced by several other factors, including the immune reaction to neoplastic cells and the consequent chronic inflammation typically observed in patients affected by advanced cancer [158]. Therefore, while the association between plasmatic cfDNA levels and tumor stage has been thoroughly described over the years, many confounding factors pose serious limitations for its assessment as a prognostic biomarker in patients’ early and late management. On the other hand, given its higher specificity, the detection and quantification of ctDNA has a more solid potential for the prognostic stratification of patients affected by solid tumors in the early phases of their management [158]. High levels of ctDNA in plasma are in fact associated with a worse overall survival in patients affected by the most common solid tumors. However, besides all the technical difficulties typically encountered for the assessment of ctDNA, a certain degree of heterogeneity is reported alongside diverse studies with regard to the impact of ctDNA detection on the prognosis of neoplastic patients. Moreover, while the clinical implications of ctDNA assessment for the early detection of MRD or actionable mutations in the bloodstream are well recognized, patients’ prognostic stratification based on ctDNA assessment may not bring significant advancements in their clinical management, as a global consensus is required for the elaboration of specific thresholds for clinically valid prognostic classes.

## 8. Theranostic Applications of cfRNA Assessment

The assessment of cfRNA has been studied, even though with less successful results, for virtually all ctDNA applications, from early diagnosis, to prediction of recurrence, prognostic stratification and prediction of response/resistance to treatment [159,160]. In addition to the analysis of gene expression, which is typically regarded as the main application of RNA-based biomarkers, cfRNA evaluation allows the detection of fusion events, alterations in splicing and post-transcriptional processes of transcript modification, besides transcriptional patterns informative of tissue origin.

As previously described, RNA-based biomarkers include both coding and non-coding transcripts, with diverse implications regarding their possible use for clinical purposes. Of the manifold applications for which coding cfRNA has been investigated, an increasing interest toward its theranostic applications is documented. In particular, the assessment of transcriptional alterations in circulating RNA may allow a targeted non-invasive directioning of the systemic treatment, with particular concern to NSCLC. A fraction of patients affected by NSCLC benefit from specific targeted therapies, depending on the specific transcriptional alterations. Some of these therapies are already FDA approved for a specific subset of patients, for example, those affected by NSCLC harboring fusion genes of ROS1 or ALK, while others are still in the phase of transition into clinical practice with effective results in clinical trials. Diverse protocols have been developed for the assessment of circulating fusion transcripts, including ALK, ROS1 and RET with a coverage of 100, 88 and 99%, respectively, of the diverse fusion variants, while NTRK fusions are suboptimally covered by current RNA-based NGS panels [89,161,162]. The assessment of cfRNA for theranostic purposes may include the detection of alterations in alternative splicing, reported as associated either with the acquisition of resistance to systemic treatment or response to targeted therapy. For example, RNA-based assays seem to overcome DNA sequencing for the assessment of MET exon 14 skipping, even though this does not apparently correlate with the response to MET tyrosine kinase inhibitors [163].

## 9. Conclusions

The assessment of cfNAs has great potential to enter clinical practice as a novel and minimally invasive approach for the detection and monitoring of cancer through several body fluids instead of tumor tissue. The current main limitation of liquid biopsy is that it can be more appropriately defined as a field of research rather than a methodology, and as such a forest of methodologies and analytes must be thoroughly explored before a single, most promising tree is found. It is likely that several incremental technological advances over the next few years, combined with large, prospective, well-designed clinical trials devised to answer specific, clinically useful questions—such as early diagnosis, detection of MRD or identification of selected therapeutic targets in early or advanced disease—will ultimately lead to routine applications of some of the several approaches to liquid biopsy introduced in the present review.

## Figures and Tables

**Table 1:** Most common commercially available collection tubes for cfNAs preservation. For new products, no evaluation/comparison studies have been published to date (*). NA: not available.

Specimen	Collection Tubes	Storage Time and Temperature	Type of cfNAs	Ref.
Blood	cell-free DNA BCT (Streck, Omaha, NE, USA)	Up to 7 days 18 to 25 °C	cfDNA	[22]
	RNA Complete BCT (Streck, Omaha, NE, USA)	Up to 7 days 18 to 25 °C	cfRNA, exosomes	[23]
	PAXgene Blood ccfDNA (PreAnalytiX GmbH, Hombrechtikon, Switzerland)	Up to 7 days at RT (15–25 °C). Up to 24 h at 35 °C	cfDNA	[24,25]
	Cell-Free DNA Collection (Roche Diagnostics, GmbH, Mannheim, Germany)	Up to 7 days at RT	cfDNA	[23]
	Norgen Biotek cfDNA Preservative (Norgen Biotek, Corp., Thorold, ON, Canada)	cfDNA 30 days at RT and for up to 8 days at 37 °C. cfRNA for 30 days at RT.	cfDNA, cfRNA	[26]
	ImproGene Cell Free DNA (Improve, Instruments Co., Ltd., Guangzhou, China)	7–14 days under 4–30 °C	cfDNA	*
	Biomatrica LBgard Blood (Biomatrica, Inc., San Diego, CA, USA)	Up to 7 days under 4–25 °C. Up to 24 h at 37 °C	cfDNA	[27]
	Blood Stasis TM 21-ccfDNA (Mabio Genomics, Inc., Gaithersburg, MD, USA)	Up to 3 days at RT	cfDNA	[6]
	NICE^®^ Check cfDNA (EONE-Diagnomics, Genome Center, Incheon, Korea)	NA	cfDNA	*
	Blood Exo DNA ProTeck (ProTeck, CFGenome LLC, Denver, CO, USA)	4, 22 and 30 °C for 21, 28 and 7 days,	cfDNA	[28]
	CEE-Sure TM BCT (Biocept, San Diego, CA, USA)	cfDNA up to 8 days at under 6–37 °C. cfRNA for 30 days at RT. CTCs for 14 days at RT	cfDNA	[29]
Urine	Norgen Biotek Urine Preservative (Norgen Biotek, Corp., Thorold, ON, Canada)	2 years at RT	cfRNA, microRNA, DNA, RNA, proteins	[30]
	Cell-Free DNA Urine Preserve (Streck; Omaha, NE, USA)	Up to 7 days when stored at 6 to 37 °C.	cfDNA	[31]
	Urine collection (Human UPSBio Inc., Atlanta, GA, USA)	Up to 7 days at RT	cfDNA	[32]
	Urine Conditioning Buffer UCB (Zymo Research, Irvine, CA, USA)	1 months at RT	DNA, RNA, cfDNA	*
Saliva	Saliva Exosome Collection and Preservation Kit (Norgen, Biotek, Corp., Thorold, ON, Canada)	2 years at RT	Exosomes, RNA	[33]
	Pure SAL ^TM^ (Oasis Diagnostics, Vancouver, WA, USA)	NA	cfDNA, cfRNA, exosomes, proteins	[34]
	RNA ProSAL ^TM^ (Oasis Diagnostics, Vancouver, WA, USA)	NA	RNA, cfDNA, cfRNA, exosomes	[34]

**Table 2:** Summary of cfNAs technical applications and methods of analysis.

Analysis	Analyte	Approaches	Methods	
Mutations: point mutation, indels, amplifications, CNVs, deletions, translocations	cfNAs	Single-molecule	NGS	INC-Seq Nanopore; Pacific BioSciences PacBio
		Single-gene	PCR	qPCR; ARMS-PCR; COLD-PCR; bi-AP; MAP; ddPCR; BEAMing
		Gene panels	NGS	TAm-Seq; Safe-SeqS; SiMSen-Seq; CypherSeq; DuplexSeq; smMIPs; BAsE-Seq; CAPP-Seq; mFAST-SeqS; mmPCR-NGS;RealSeqS; bTMB assay
		Genome	NGS	WGS; WES; low-pass sequencing
		Other	spectroscopy	MALDI; SERS; UltraSEEK
Transcriptome quantification	miRNA, cf-mRNA, lncRNAs, ncRNAs	Few transcripts	PCR	qRT-PCR
		Multi-transcripts	Hybridization	Microarray
		68 target miRNAs	Flow cytometry	FireFly
		exome	NGS	Whole-transcriptome RNA-sequencing
Epigenetic modification	cfMeDNA	Single-molecule	NGS	Nanopore
		Specific CpG site	PCR	Sodium bisulfite; MSP; qMSP; EpiTect MethyLight PCR; SYBR Green-based qMSP
		All CpG sites	NGS	MethylCap; cfMeDIP-seq; EM-Seq; TAPS; cfNOMe

## Supplementary Materials

The following are available online at https://www.mdpi.com/article/10.3390/cancers13143460/s1, Table S1: Most common available extraction kits for cfNAs.
