# Supplementary material for: Assessment of Circulating Nucleic Acids in Cancer: From Current Status to Future Perspectives and Potential Clinical Applications
Source: Cancers (Basel). 2021 Jul 10;13(14):3460. doi: 10.3390/cancers13143460 (PMC8307284; doi:10.3390/cancers13143460)
Supplement: Supplementary file 1 [file cancers-13-03460-s001.zip › cancers-1239513-supplementary.pdf]

cancers

MDPI

Supplementary Material

Assessment of Circulating Nucleic Acids in Cancer: From Current Status to Future Perspectives and Potential Clinical Applications

Gabriella Cirmena, Martina Dameri, Francesco Ravera, Piero Fregatti, Alberto Ballestrero and Gabriele Zoppoli

Table S1. A list of the most common extraction kits for cf/ctDNA and cfRNA with their processing features. For sample types P = plasma, S = serum, U = urine, O = other body fluids.

Extraction Kit Name	Company	Sample Type	Method	Execution	Input vol. (mL)	Elution Vol. (µL)	Extracted Specimen
ctDNA Purification Kit	Abnova	P, S, U, O	Magnetic beads	Manual/automated protocols	2–6	50	cfDNA
Cell-Free DNA Purification Kit	Active Motif	P, S	Magnetic beads	Manual/automatable	0.1–10	6.25–50	cfDNA
Hipro Circulating Cell–Free DNA (cfDNA) Isolation Ki	Amsbio	P, S, U, O	Spin columns	Manual	0.1–1	20–100	cfDNA
PME free–circulating DNA Extraction Kit	AnalitikJena	P, S, U	Polymer mediated enrichment technology	Manual	1–10	50	cfDNA
MagMAX Cell–Free DNA Isolation Kit	Applied Biosystems	P, S, U	Magnetic beads	Manual/automated protocols	0.1–10	15–150	cfDNA
MagMAX Cell–Free Total Nucleic Acid Isolation Kit	Applied Biosystems	P	Magnetic beads	Manual/automated protocols	1–6	15–60	cfDNA/RNA
Apostle MiniMax High Efficiency Isolation Kit	Beckman Coulter	P, S, U	Magnetic beads	Manual/automatable	1–5	2–100	cfDNA
cfPure Cell Free DNA Extraction Kit	BioChain	P, S, U	Silica–coated paramagnetic particles	Manual/automated protocols	<1–10	15–50	cfDNA
MagListo cfDNA Extraction Kit	Bioneer	P, S, U, O	Magnetic nanobeads	Manual/automated protocols	1–5	30–70	cfDNA
NextPrep–Mag cfDNA Isolation Kit	Bioo Scientific	P	Magnetic beads	Manual or automated version	1–5	12–60	cfDNA
NextPrep–Mag Urine cfDNA Isolation Kit	Bioo Scientific	U	Magnetic beads	Manual, automation–friendly	1–20	3–60	cfDNA
The NextPrep Magnazol cfRNA Isolation Kit	Bioo Scientific	P, S	Magnetic beads	Manual	0.2–1.2	<18	cfRNA
SubXTM cfDNA Isolation kit	Capital Bioscences	P, S, U	SubX substance and solid phase matrix	Manual	2–50	20–50	cfDNA
truXTRAC cfDNA Kit	Covaris	P	Magnetic beads	Manual/automated protocols	1	35–50	cfDNA
Sera–Xtracta Cell–Free DNA Kit	Cytiva	P, S, U	Magnetic beads	Manual/automated protocols	0.5–4	15–60	cfDNA

Helix Circulating Nucleic Acids Kit	Diatech pharmacogenetics	P	Silica membrane system	Manual	1–5	ND	cfDNA
EpiQuik Circulating Cell–Free DNA (ccfDNA) Isolation Easy Kit	Epigentek	P, S, U, O	Magnetic beads	Manual	0.1–1	20	cfDNA
Presto cfDNA/cfRNA Extraction Kit	Geneaid	P, S	Spin columns	Manual	1–5	30–50	cfDNA/RNA
cfDNA/RNA Extraction Kit	Ibi Scientific	P, S	Spin columns	Manual	1–5	30–50	cfDNA/RNA
NucleoSpin cfDNA Kits	Macherey–Nagel	P, S	Silica membrane columns	Manual (vacuum)	0.2–5	100/200	cfDNA
NucleoSnap cfDNA Kit	Macherey–Nagel	P, S, U	Silica membrane columns	Manual (vacuum)	1–10	20–100	cfDNA
NucleoSpin cfDNA XS	Macherey–Nagel	P	Silica membrane columns	Manual (centrifugation)	<0.240	5–30	cfDNA
cfKapture Kit	Magbio	P, S	Magnetic beads	Manual	0.2–5	30–100	cfDNA
GenElute UltraMag Cell–Free DNA Kit	Merck	P	Magnetic beads	Manual	0.5–10	6–125	cfDNA
GenElute Urine Cell–Free DNA Purification Kit	Merck	U	Spin column chromatography	Manual	0.250–2	25–100	cfDNA, viral DNA
Plasma/Serum Cell–Free Circulating DNA Purification Kits	Norgen Biotek Corp	P, S	Spin column chromatography	Manual	0.01–10	25–50	cfDNA, bacterial or viral DNA
Plasma/Serum cfc–DNA Advanced Purification Kit	Norgen Biotek Corp	P, S	Spin column chromatography	Automated (Hamilton MicroLab Nimbus)	0.5–6	25–50	cfDNA
Urine Cell–Free Circulating DNA Purification Kits	Norgen Biotek Corp	U	Spin column chromatography	Manual	0.250–30	50–100	cfDNA, viral DNA
Mag–Bind cfDNA Kit	Omega Biotek	P, S	Magnetic beads	Manual/automated protocols	0.5–10	50	cfDNA
Phasify MAX cfDNA Extraction Kit	Phasify	P	Liquid phase mechanism	Manual	01–feb	5–150	cfDNA
Maxwell RSC ccfDNA Plasma Kit	Promega	P	Magnetic beads	Automated	0.2–1	<60	cfDNA
QIAamp Circulating Nucleic Acid	Qiagen	P, S, U, O	Silica membrane columns	Manual (vacuum)	1–5	20–150	cfDNA/RNA, miRNA, viral DNA/RNA
QIAsymphony DSP Circulating DNA Kit	Qiagen	P, U	Magnetic beads	Automated (QIAsymphony SP)	2/4	60	cfDNA
QIAamp DSP Circulating NA Kit	Qiagen	P, S, U	Silica membrane columns	Manual (vacuum)	1–5	20–150	cfDNA/RNA/miRNA
QIAamp MinElute ccfDNA Kit	Qiagen	P, S	Magnetic beads	Partially automated	1–10	20–80	cfDNA
QIAamp ccfDNA/RNA Kit	Qiagen	P, S	Silica membrane columns	Partially automated	1–4	14–20	cfDNA/RNA, miRNA, vesicular nucleic acids
EZ1 ccfDNA Kit	Qiagen	P, S	Magnetic beads	Semi–automated	1–10	55–65	cfDNA
miRNeasy Serum/Plasma Kit	Qiagen	P, S, O	Spin columns	Manual/automatable	<0.2	14	cfRNA, miRNA

MagNA Pure 24 Total NA Isolation Kit	Roche	P	Magnetic glass particles	Automated (MagNA Pure 24 System)	2/4	100/150/200	Single or double stranded cfDNA
Sentosa SX Cell–free DNA Kit	Vela Diagnostics	P	Magnetic beads	Automated	>4	ND	cfDNA
IDXtract–MAG	Xid Solutions	P	Magnetic beads	Automated	1–8	ND	cfDNA
Quick–cfDNA/cfRNA Serum & Plasma Kit	Zymo Research	P, S, U, O	Zymo–Spin IC Columns	Manual	0.2–3	6–15	cfDNA/RNA
